# Sleep, PTSD, and suicide risk in U.S. veterans

**DOI:** 10.3389/frsle.2026.1837234

**Published:** 2026-06-25

**Authors:** Sara Kintzle, Leonidas Marin, Eva Alday, Nicholas Barr

**Affiliations:** 1 Suzanne Dworak-Peck School of Social Work, University of Southern California, Los Angeles, Los Angeles, CA, United States; 2 Teachers College Columbia University, New York, NY, United States; 3 School of Social Work, University of Nevada, Las Vegas, NV, United States

**Keywords:** mental health, military, sleep issues, suicide, veterans, well-being

## Abstract

**Introduction:**

The stresses of military service can contribute independently to mental health and sleep problems in military personnel that persist after leaving military service. Understanding how sleep and mental health challenges contribute to behavioral health outcomes is key to supporting this population. To advance these goals, this study examined associations among PTSD, sleep disturbance, and suicide risk in U.S. veterans.

**Methods:**

Online survey data using valid and reliable measures were collected from3,188 veterans living in Southern California as part of a large needs assessment; suicide risk was measured by the SBQ-R, which assess ideation, behaviors and attempts. A structural equation modeling approach was used to test the direct effects of PTSD on sleep problems and suicide risk, the direct effect of sleep on suicide risk, and an indirect effect of PTSD on suicide risk through the sleep pathway.

**Results:**

Findings demonstrated significant direct effects for PTSD (B = 0.05, *p* < 0.001) and sleep problems (B = 0.74, *p* < 0.001) on suicide risk, as well as a significant indirect effect for PTSD on suicide risk through the sleep pathway (B = 0.05, *p* < 0.05). The structural model explained 55.3% of the variance in sleep difficulties and 29.9% of the variance in suicide risk.

**Discussion:**

Results advance the understanding of how sleep problems may be associated with the risk of suicide in U.S. veterans, both directly through its effects on suicide risk and indirectly through the effects on PTSD symptomatology. Practitioners and policy makers may consider ensuring the assessment and treatment of sleep problems in veterans are included in suicide prevention models.

## Introduction

Sleep is a critical process for physical, mental, and emotional functioning, and is considered one of the three pillars of good health, alongside nutrition and physical activity (Brewer-Smyth, 2022). The U.S. Department of Health and Human Services' (HHS) Office of Disease Prevention and Health Promotion lists sleep as one of the objectives of the Healthy People 2030 initiative (U.S. Department of Health and Human Services, n.d.). High quality sleep is critical for cognitive function, overall brain health (Tai et al., 2022), and well-being (Lee and Lawson, 2021), yet the global prevalence of sleep disturbance (i.e. insomnia, nightmares, breathing disorders, and sleep duration) continues to be a serious public health concern, with estimates of disturbed sleep among adults ranging from 20 to 30 percent of the population (Di et al., 2022; Chattu et al., 2018). In military populations, prevalence rates of sleep issues have been found to be significantly higher than their civilian counterparts. For example, a recent systematic review and meta-analysis found that the global pervasiveness of poor sleep in military personnel and veterans was 69% based on 58 eligible studies of 28,100 participants (Bai et al., 2023). Given the criticality of sleep for overall functioning and well-being, more understanding is needed regarding the experiences and implications of poor sleep-in military veterans.

Due to the inherent demands of their profession, military personnel are more likely to experience a number of conditions that contribute to the development of sleep problems (Good et al., 2020). Unpredictable sleep routines, dangerous environments, exposure to trauma, suboptimal sleeping conditions, including constant noise and concern for family at home, rank among the major contributors to poor sleep quality in military personnel (Bai et al., 2023; Peterson et al., 2008). Indeed, sleep problems in service members were known to persist upon return home after the most recent conflicts in Iraq and Afghanistan, where service members in all branches of the military saw increases in sleep disorders (Colvonen et al., 2020; Bai et al., 2023). Additionally, research with veterans using VHA primary care has increasingly demonstrated significant sleep problems in this cohort following post-military transition (Byrne et al., 2021; Bonfils et al., 2023).

The extant literature has shown sleep difficulties to be highly comorbid with many psychiatric disorders, including posttraumatic stress disorder (PTSD) (Atwood, 2022), which lists sleep deficiency as a diagnostic criterion (American Psychiatric Association, 2013). Estimates of sleep problems in individuals diagnosed with PTSD range from 50 to 91%, making it a “hallmark” symptom (Milanak et al., 2019; Bai et al., 2023). Poor sleep quality is among the most commonly occurring symptoms of PTSD and is more than three times greater among veterans who screened positive for PTSD compared with those who did not (McCarthy et al., 2019). In their longitudinal study of combat-exposed veterans, Rosen et al. (2019) found that of those in their sample who reported nonrestorative sleep, a significant proportion were from veterans with PTSD diagnoses. While sleep problems have been commonly marked as a result of PTSD, more recent studies have shed light on the bidirectional relationship between PTSD and sleep problems (Slavish et al., 2022; Messman et al., 2023), where sleep problems may be a risk factor for developing and worsening PTSD. Miller et al. (2020), for example, found that lower quantities of sleep in military service members before deployment were related to developing post deployment PTSD, while DeViva et al. (2021) reported that veterans who rated their sleep quality as poor at baseline had a 60% increased risk for developing PTSD over the following 7 years. As such, sleep problems are not only a symptom of PTSD—when present prior to trauma exposure, they may also be a contributing factor in the development of PTSD (Weber et al., 2020). It is also important to note that sleep disturbance often persist after successful PTSD treatment (Pruiksma et al., 2016).

Additionally, a substantial body of literature has identified both PTSD and sleep disturbances as unique contributors to suicidality. Sleep problems are thought to impair brain function, disrupt emotional regulation and caused increased hopelessness, which can lead to suicide risk (Bernert and Joiner, 2007). Research has shown a positive association between PTSD and suicidality (Panagioti et al., 2012; Pompili et al., 2013). Military veterans, in particular, experience suicidal ideation to a greater degree compared to non-veterans (Kumar et al., 2024), with a recent study reporting that veterans with subjective cognitive difficulties and PTSD were six times more likely to report suicidal ideation (Cations et al., 2024). Sleep problems have also been identified as a risk factor for suicidality (McCarthy et al., 2022; Pigeon et al., 2012). However, the relationship between the two is mixed, as some investigations report statistically significant yet weak associations (Harris et al., 2020; McCarthy et al., 2022), while others have detected more robust association between sleep disturbance and suicide risk (Batterham et al., 2021; Liu et al., 2020). In a study comparing veteran decedents of suicide, those with documented sleep disturbances died sooner after their last medical visit than those without sleep disturbance (Pigeon et al., 2012), highlighting how the presence of sleep problems may be useful in detecting risk for suicide.

These findings underscore the growing importance of studying the associations between PTSD, sleep problems, and suicidality, as veterans with PTSD and other comorbid disorders have a higher risk of suicidal ideation than those with PTSD only (Jakupcak et al., 2009). For example, a recent study found high prevalence of current suicidal ideation and lifetime suicide attempts in individuals with comorbid PTSD and insomnia (Georgescu et al., 2024). PTSD symptoms have also been found to have an indirect effect on suicidality through sleep disturbances in civilian samples (Betts et al., 2013), while a longitudinal study looking comorbid PTSD, sleep problems, and chronic pain found that sleep disturbances were prevalent in 90% of veterans up to 15 months after their discharge from the military, and were linked to greater risk for suicidal ideation (Shor et al., 2023). In spite of the growing number of investigations targeting associations between sleep, PTSD, and suicide risk, there is little research examining direct and indirect effects of PTSD and sleep disturbance on suicide risk in community dwelling U.S. veterans. The purpose of the present study was to conduct such an investigation.

## Methods

### Participants and procedures

Online surveys via the Qualtrics platform were used to collect data from July 2022 to June 2023. This effort was part of large regional needs assessment in three counties located in Southern California (Kintzle et al., 2023). The study team employed multiple recruitment strategies to achieve maximum representativeness of the veteran population. The research team partnered with local and regional veteran collaboratives and veteran service organizations (VSOs) in the target area. Partners used their contact databases to email potential participants the survey invitation and/or share the opportunity on their social media platforms. Additionally, national veteran organizations were leveraged to identify Southern California veterans from their email lists. The final recruitment strategy employed print and social media advertisements to build a presence within the Southern California community. Social media platforms, such as Instagram, X (Twitter), Facebook and LinkedIn, along with the study website and mass emails, promoted the research opportunity to potential participants in Southern California. Once potential participants were identified, they were sent an email invitation that included the study informed consent, and a link to complete the survey. The survey took approximately 30–60 min to complete, and all participants received a $25 gift card for completing their survey. All data collection procedures were approved by the (blinded for review) Institutional Review Board and informed consent was obtained from all participants.

### Measures

#### Demographic characteristics

Sample demographics including age, gender, race, marital status, level of education, branch of service, deployment experience, and discharge status, were assessed using categorical self-report items.

#### Posttraumatic stress disorder

PTSD was measured using the PTSD Checklist for DSM-5 Short Form (PCL-5; Zuromski et al., 2019). Developed by the National Center for PTSD as a shortened version of the PCL-5, this self-report questionnaire assesses the presence and severity of post-traumatic stress disorder (PTSD) symptoms according to the Diagnostic and Statistical Manual of Mental Disorders, 5th Edition (DSM-5) criteria. The measure comprises four items, which ask respondents how much they have been bothered by PTSD symptoms in the last month (e.g., “*feeling distant or cut off from other people*”; “*Feeling irritable or angry or acting aggressively”; Avoiding external reminders” and “suddenly feeling or acting as if the stressful experience were happening again”*) with responses choices ranging from 0 (not at all) to 4 (extremely). Total scores range from 0 to 16 (Costa et al., 2023). Higher scores indicate more symptoms; a score of 7 was chosen to indicate probable PTSD utilizing supplemental diagnostic threshold material provided by (Zuromski et al. 2019), as the score provided both diagnostic sensitivity and specificity. This cutoff score has also been used by previous authors (Costa et al., 2023). The measure had strong reliability with a Cronbach's alpha of 0.89.

#### Sleep

The Insomnia Severity Index (ISI) was used to measure sleep problems (Bastien et al., 2001). This brief, self-report questionnaire assesses the severity of insomnia symptoms and the impact of insomnia on daily functioning. The ISI can be useful in identifying individuals who may be experiencing insomnia symptoms, and in monitoring the effectiveness of treatments aimed at reducing insomnia symptom severity and improving daytime functioning. The measure consists of 7 items related to sleep behaviors and satisfaction (e.g. “*How satisfied/dissatisfied are you with your current sleep pattern?”, “To what extent do you consider your sleep problem to interfere with your daily functioning, currently?”*). Response options vary by item, with a total scale range of 0–28. Higher scores represent more sleep problems, with scores above 15 indicating clinically significant insomnia (Bastien et al., 2001). The measure had strong reliability with a Cronbach's alpha of 0.92.

#### Suicide risk

Participants' suicide risk was measured using The Suicide Behaviors Questionnaire-Revised (SBQ-R; Osman et al., 2001). This self-report questionnaire asks about suicide ideation in both lifetime and the previous year, disclosure of suicide ideation, and perceived likelihood of suicide attempt. Examples of items include “*have you ever thought about or attempted to kill yourself”* and “*have you ever told someone that you were going to commit suicide, or that you might do it.”* Response options vary by item, with a total scale range of 3–18. Higher scores indicate more risk, with a score of 7 or above indicating clinically significant suicide risk (Osman et al., 2001). The measure had strong reliability with a Cronbach's alpha of 0.81.

### Data analyses

To characterize the sample, descriptive statistics and frequencies were first computed for demographic, military background variables, and the key study variables of PTSD, sleep, and suicide risk. Next, zero-order Pearson correlations among PTSD symptom severity, sleep problems, and suicide risk were computed to describe bivariate associations among study variables prior to structural equation modeling. We then applied a structural equation modeling approach to test direct effects of PTSD on sleep problems and suicide risk, a direct effect of sleep on suicide risk, and an indirect effect of PTSD on suicide risk through the sleep pathway. We selected a latent modeling approach over the use of composite or summary scores for several reasons. First, latent factors explicitly account for measurement error in each indicator, whereas composite scores treat all variance as true score variance, potentially attenuating or inflating path coefficients (Kline, 2023). Second, although the PCL-5 Short Form, ISI, and SBQ-R are brief measures, brevity does not preclude latent factor estimation when the indicator-to-factor ratio is adequate and model identification criteria are met, conditions satisfied in the present model. Third, the use of latent variables permits the simultaneous estimation of measurement and structural parameters, providing a more rigorous test of the hypothesized relationships among study variables than would be possible with observed composite scores.

Measurement models for PTSD, sleep problems, and suicide risk latent factors were specified using all observed indicators for the respective factor scales and estimated simultaneously with direct and indirect effects among latent factors in MPLUS version 8. The first observed indicator loading for each factor was fixed at 1 to establish a reference point for latent variable scales. The estimator was maximum likelihood, and standard errors were bootstrapped with 1,000 iterations. Consistent with best practices (West et al., 2012), overall model fit was assessed using model CFI, TLI, and RMSEA indices, as well as substantive utility. Missing data were handled using full information maximum likelihood (FIML) estimation, which retains all available cases by using all observed data to inform parameter estimates.

## Results

### Sample demographics

In total, 3,188 veterans participated in the study and completed the survey. The majority of the sample were white (43.3%), male (81.9%), and were married or had a long-term partner (57.5%). Slightly over forty percent of the sample (41.8%) had a college degree, and the average age was 49.16 (SD = 16.09). The Army (35.2%) was the most represented branch of the military followed by the Navy (28.7%), Marine Corps (21.5%), and Air Force (11.6%). Most participants reported being active duty at the time of separation or retirement from the military (73.8%) and had been deployed during their service (76.3%). All demographic descriptive information can be found in Table 1. Mean scores, standard deviations and the sample percentage meeting clinically significant thresholds for model variables PTSD, sleep and suicide risk can be found in Table 2. Zero-order correlations among PTSD symptom severity, sleep problems, and suicide risk revealed significant positive associations among all three constructs; PTSD symptom severity was strongly correlated with sleep problems (*r* = 0.69, *p* < 0.001) and moderately correlated with suicide risk (*r* = 0.47, *p* < 0.001). Sleep problems were also significantly associated with suicide risk (*r* = 0.39, *p* < 0.001).

**Table 1:** Sample demographics.

Demographic characteristics	Means (SD) frequencies (%)
Age	49.16 (16.09)
Race	
White	1,378 (43.3)
Black or African American	373 (11.7)
Hispanic, Latino, or Spanish	295 (9.3)
Asian	307 (9.6)
Other race	830 (26.0)
Gender	
Male	2,556 (80.7)
Female	562 (17.7)
Gender diverse	49 (1.5)
Marital status	
Married/partner	1,831(57.5)
Divorced/separated/widow(er)	597 (18.8)
Single	755 (23.7)
Education	
High school degree	1,143(35.9)
College degree	1,331 (41.8)
Graduate degree	633 (19.9)
Other education	79 (2.5)
Branch	
US Air Force	370 (11.6)
US Army	1123 (35.2)
US Coast Guard	90 (2.8)
US Marine Corps	686 (21.5)
US Navy	915 (28.7)
US Space Force	1 (0.0)
Deployed	
Yes	2433 (76.3)
No	741 (23.2)
Missing	14 (0.4)
Discharge status	
Active	2353 (73.8)
Reserve	636 (19.9)
National guard	168 (5.3)

**Table 2:** Means and standard deviations of SEM variables.

Variable	Mean (SD)	% meet clinically significant cutoff
PCL-5 SF (PTSD)	5.58 (4.65)	38.9%
ISI (sleep problems)	10.91 (7.32)	31.1%
SBQ-R (suicide risk)	5.27 (2.94)	26.3%

### Measurement model and direct and indirect effects

**Figure 1:**
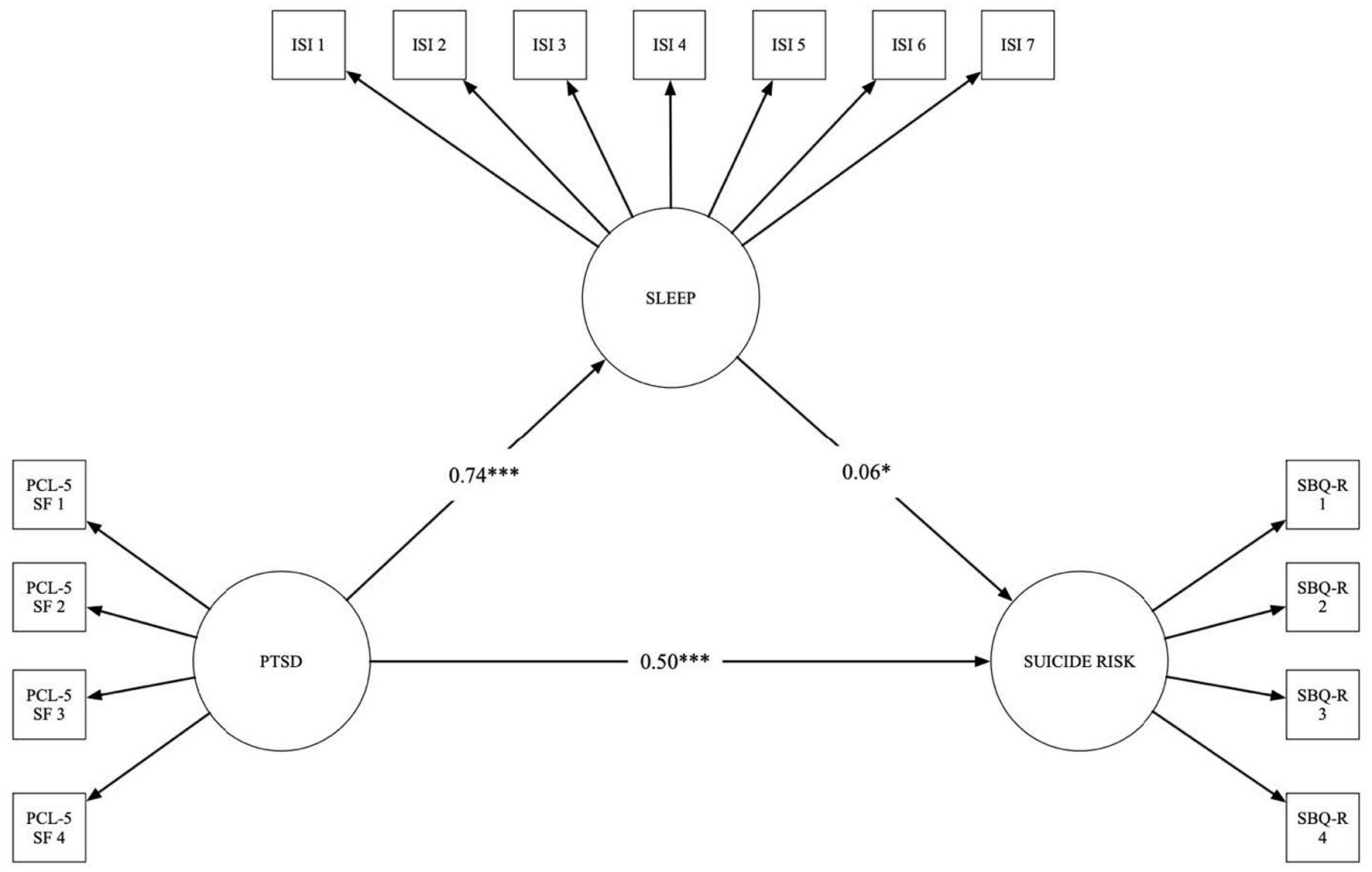
Structural equation model with standardized estimates for associations among latent factors. *p < 0.05; **p < 0.01; ***p < 0.001. Sleep refers to the sleep difficulties latent factor derived from Insomnia Severity Index (ISI) items 1–7. PTSD refers to the posttraumatic stress disorder latent factor derived from Posttraumatic Stress Disorder Checklist for DSM-5 short form (PCL-5 SF) items 1–4. Suicide Risk refers to the latent factor derived from Suicide Behaviors Questionnaire, Revised (SBQ-R) items 1–4.

The analytic sample included 3,175 veterans who had complete data on all model variables. Results showed good model fit to the data, with (*X*^2^ = 1,941.70, df = 87), RMSEA = 0.08, CFI = 0.94, and TLI = 0.93. All latent factor loadings were significant at the *p* < 0.001 threshold. Examination of standardized estimates showed significant direct effects for PTSD on suicide risk (*B* = 0.50, *p* < 0.001) and sleep problems (*B* = 0.74, *p* < 0.001), and sleep problems on suicide risk (*B* = 0.06, *p* < 0.05). The indirect effect of PTSD on suicide risk through sleep disturbance was small but statistically significant [*B* = 0.05, 95% CI (0.003, 0.085)], based on 1,000 bootstrap samples. The structural model accounted for 55.3% of the variance in insomnia severity (*R*^2^ = 0.553, *SE* = 0.017, *p* < 0.001) and 29.9% of the variance in suicide risk (*R*^2^ = 0.299, *SE* = 0.018, *p* < 0.001). Standardized and unstandardized estimates for factor loadings and direct and indirect effects among latent factors are shown in Table 3. The structural equation model with standardized estimates for associations among PTSD, sleep, and suicide risk latent factors is shown in Figure 1.

**Table 3:** Standardized and unstandardized parameter estimates for latent factor loadings, and direct and indirect effects.

Estimates	Standardized	Unstandardized	S.E.	*P*-value
Factor loadings				
**PTSD**				
PCL-5 SF item 1	0.78	1.00	0.00	< 0.001
PCL-5 SF item 2	0.85	1.21	0.02	< 0.001
PCL-5 SF item 3	0.85	1.21	0.03	< 0.001^***^
PCL-5 SF item 4	0.79	1.07	0.03	< 0.001^***^
**Sleep problems**				< 0.001^***^
ISI item 1	0.81	1.00	0.00	< 0.001^***^
ISI item 2	0.82	1.01	0.01	< 0.001^***^
ISI item 3	0.57	0.62	0.02	< 0.001^***^
ISI item 4	0.82	0.96	0.02	< 0.001^***^
ISI item 5	0.80	0.98	0.02	< 0.001^***^
ISI item 6	0.89	1.06	0.02	< 0.001^***^
ISI item 7	0.88	1.09	0.02	< 0.001^***^
**Suicide risk**				< 0.001^***^
SBQ-R item 1	0.79	1.00	0.00	< 0.001^***^
SBQ-R item 2	0.78	1.11	0.04	< 0.001^***^
SBQ-R item 3	0.61	0.42	0.02	< 0.001^***^
SBQ-R item 4	0.76	1.16	0.04	< 0.001^***^
Direct effects				
**PTSD**				
Suicide risk	0.50	0.36	0.03	< 0.001^***^
Sleep problems	0.74	0.80	0.03	< 0.001^***^
**Sleep problems**				
Suicide risk	0.06	0.04	0.02	< 0.05^*^
Indirect effect				
PTSD on suicide risk	0.05	0.03	0.02	< 0.05^*^

^*^p < 0.05. ^**^p < 0.01. ^***^p < 0.001.

## Discussion

The purpose of this study was to examine the direct effects of PTSD on sleep problems and suicide risk, the direct effects of sleep problems on suicide risk, and the indirect effect of PTSD on suicide risk through the sleep pathway. Results advance the understanding of how addressing sleep problems may be associated with the risk of suicide in U.S. veterans, both directly and indirectly through their effects on PTSD symptomatology.

As expected, PTSD had a significant direct effect on suicide risk, demonstrating that individuals experiencing PTSD symptoms had higher risk for suicide. This finding is consistent with previous research demonstrating PTSD as a risk factor for suicidal thoughts and behaviors in the veteran population (Akbar et al., 2023; Schafer et al., 2021). These results support current recommendations around the inclusion of early identification and treatment of PTSD in suicide prevention programs. Recent literature has demonstrated that reductions in suicide risk are associated with PTSD treatment even when suicidality is not an explicit treatment target (Pease et al., 2023; Saulnier et al., 2024). Study findings support the continued use of resources that work to increase access to mental health care, decrease stigma of such care, and improve mental health treatment outcomes for veterans.

Sleep problems were also found to have a direct effect on suicide risk, with veterans in the sample experiencing sleep problems more likely to be at risk for suicide. It is important to note that this was a direct relationship, independent of PTSD symptoms which may also contribute to sleep disturbance. This finding aligns with previous literature that has demonstrated associations between sleep and suicide risk in veterans (McCarthy et al., 2022; Bishop et al., 2020). Despite these links, relatively little attention has been given to the importance of assessing and treating sleep difficulties as part of veteran suicide prevention efforts compared with efforts to identify and treat mental health disorders. This is particularly important in the veteran population, as research highlights veterans' vulnerabilities to sleep challenges during service that may continue after transitioning out of the military. Veterans also experience a number of stressors related to military service that may impact sleep, such as symptoms related to pain, musculoskeletal injuries, physical health problems and mental health symptoms (Williamson et al., 2019; Teplova et al., 2022). This vulnerability to the development of sleep problems and the importance of sleep to overall well-being, as well as its association to suicide risk, demonstrate it may be important to address sleep challenges in the development and implementation of suicide prevention efforts.

Supporting emerging literature was the finding of the indirect effect of PTSD on suicide risk through the sleep pathway (Betts et al., 2013; Rohr et al., 2021). Though small, this effect suggests that the impact of PTSD symptomatology on suicide risk is heightened in veterans who are also experiencing significant sleep issues. Hyperarousal theory offers one explanatory framework: PTSD-related hyperarousal may chronically disrupt sleep architecture, and persistent sleep disturbance may independently amplify suicidal ideation through fatigue-driven cognitive constriction and diminished problem-solving capacity (Germain, 2013; Norra et al., 2011). Emotion dysregulation models offer a complimentary explanation, suggesting that sleep loss degrades prefrontal inhibitory control, reducing the capacity to modulate trauma-related affect and thereby increasing suicide risk (Ward-Ciesielski et al., 2018; Weber et al., 2020). This finding suggests that the sleep-related symptoms of PTSD, and sleep disturbance in general, may contribute to suicide risk in veterans with PTSD. These findings have important implications for both the treatment of PTSD and suicide prevention; targeting sleep disturbance directly may impact suicide risk both directly and indirectly in veterans with PTSD, thus providing a highly efficient treatment focus. However, longitudinal and experimental designs that manipulate sleep or target hyperarousal directly are needed to test whether these pathways are causal and whether sleep intervention might attenuate suicide risk in this population.

It is well established that sleep has profound impacts on health, well-being and suicide risk (Chattu et al., 2018; Grandner, 2017). These associations are the start of a framework that suggests ensuring sleep issues are addressed early in treatment may have positive effects on outcomes in veterans with PTSD. As sleep is connected to overall health, reducing or alleviating sleep problems could create quick improvements in veterans' feelings of well-being. Seeing early improvements in symptoms can keep veterans in treatment and reduce client attrition. In addition, given the impact of the sleep pathway on suicide risk, addressing sleep issues in treatment may be associated with reduced risk for dying by suicide. It is important that practitioners providing PTSD treatment to veterans have resources and interventions that specifically focus on problems with sleep. This recommendation aligns with recent findings in the literature. A meta-analysis of randomized controlled trials by Scott et al. (2021) found improvements in sleep quality led to great improvements in mental health, leading authors to recommend further research into how sleep interventions might be incorporated into mental health services.

These findings also have implications for veteran suicide prevention programs. Results indicate sleep may be a significant risk factor for dying by suicide, however, it is not routinely screened for or addressed within suicide prevention. In light of the direct and indirect impacts of sleep on suicide risk, it may be an important factor that is currently missing in prevention efforts. Given veterans' susceptibility of sleep challenges, as well as the importance of sleep in well-being, veteran service organizations may want to consider sleep screenings as part of their regular client intake form, as well as having the ability to refer to resources if needed.

As in all research, several limitations must be noted. First, the data are cross-sectional and therefore cannot support causal inferences. Longitudinal or intervention studies would be required to establish casual links between the study variables. Second, the study utilized convenience samples and a study population that consisted of veterans living in Southern California, which may limit the generalizability of the results to the broader veteran population. Third, while the sleep measure used is a well-established, valid, and reliable measure of sleep problems, it does not measure sleep quality, which is an important aspect of understanding sleep. Fourth, the study utilized self-report measures, which often have discrepancies with objective measures, particularly for the construct of sleep. Fifth, the study model focused specifically on the variables of PTSD, sleep and suicide to establish baseline associations. This means that potential confounding variables that may impact these relationships were not included in the model. Lastly, as the model being tested was exploratory, it did not include covariates that may also impact the relationship between PTSD, sleep, and suicide risk, or the bidirectional relationship between PTSD and sleep. Despite these limitations, the study findings build an important foundation for understanding the impact sleep problems may have on suicide risk in veterans with PTSD. Further research should expand this understanding by examining more complex models that include additional variables that may impact sleep, as well as exploring protentional differences in relationships between variables based on demographic characteristics. Additionally, the impact of sleep on general health should continue to be explored in the veteran population, as well as how interventions might reduce suicide risk and improve overall well-being.

## Data Availability

The original contributions presented in the study are included in the article/supplementary material, further inquiries can be directed to the corresponding author.
